# The molluscan assemblage of a pristine *Posidonia oceanica* meadow in the eastern Mediterranean

**DOI:** 10.1007/s12526-022-01292-2

**Published:** 2022-10-13

**Authors:** Martina Holzknecht, Paolo G. Albano

**Affiliations:** 1 Department of Palaeontology, University of Vienna, Althanstrasse 14, 1090 Vienna, Austria; 2 Department of Animal Conservation and Public Engagement, Stazione Zoologica Anton Dohrn, Villa Comunale, Naples, Italy

**Keywords:** Seagrass, Mollusca, Global warming, Lessepsian invasion, Crete, Levantine Basin

## Abstract

The seagrass *Posidonia oceanica* forms extensive meadows in the Mediterranean Sea. Studies on their associated highly diverse invertebrate assemblages are limited to the western Mediterranean. The eastern Mediterranean, however, is a basin undergoing rapid change due to the synergistic effects of climate warming, biological invasions and other human stressors that are driving native biodiversity to regional-scale collapses. We here surveyed the shelled molluscan assemblage of a *Posidonia oceanica* meadow in Plakias, south-western Crete, the first such study in the eastern Mediterranean Sea. This area has increased its yearly mean temperature by 1 °C in the last 20 years and is under heavy pressure by Lessepsian species. We sampled across a 5- to 20-m depth gradient, in two seasons to capture intra-annual variation and the leaf and rhizome strata separately. Against our expectations, the molluscan assemblage proved to be highly diverse, with species richness, dominant species and trophic guilds comparable to healthy western Mediterranean ones, and with a negligible non-indigenous component. The diversity of the native community (following the biotic resistance hypothesis) and oxygen supersaturation in the meadow may cause greater resistance to biological invasions and warming, respectively, suggesting that *Posidonia oceanica* meadows may act as a precious refugium for native biodiversity in the fast changing eastern Mediterranean Sea.

**Supplementary Information:**

The online version contains supplementary material available at 10.1007/s12526-022-01292-2.

## Introduction

Biological invasions and global warming are among the main pressures affecting marine biodiversity in the eastern Mediterranean Sea. After the opening of the Suez Canal in 1869, hundreds of species entered the basin and established abundant populations (Galil 2009; Zenetos et al. 2012; Nunes et al. 2014) in the so-called Lessepsian invasion. Some species have exerted major impacts on native biota. For example, rabbitfishes overgraze algae (Sala et al. 2011) and the lionfish *Pterois miles* disrupts local marine communities (Savva et al. 2020). Additionally, the seawater temperature in the Mediterranean Sea has been increasing for the last few decades, but the increase in the Aegean and Levantine Seas has been the highest in the Mediterranean reaching 0.048 ± 0.006 °C/year over 1982–2018 (Pisano et al. 2020). This abrupt increase has likely triggered the disappearance of native species from the warmest areas like the Israeli shelf (Yeruham et al. 2015; Rilov 2016; Albano et al. 2021a). In such most affected areas, biological invasions and climate warming are acting in concert substantially modifying not only taxonomic composition but also ecosystem functioning (Rilov et al. 2018; Peleg et al. 2019; Yeruham et al. 2019; Steger et al. 2021a,b).

The South Aegean is in a special position in the eastern Mediterranean Sea because it bridges the Levantine Sea, its easternmost sector and the most affected by the Lessepsian Invasion and seawater warming, with the Ionian Sea. It is among the most invaded areas in Greece (Zenetos et al. 2011). A review of non-indigenous species in Crete is not available, but the recent demographic explosion of invasive Lessepsian species such as *Pinctada radiata* and *Fistularia commersonii* (Zenetos et al. 2008; Zenetos 2015) shows that this island is heavily affected similarly to other areas in the eastern Mediterranean (e.g. Dimitriadis et al. 2020).

The Mediterranean endemic plant *Posidonia oceanica* forms meadows that host a very high biodiversity and productivity (Boudouresque et al. 2006). However, research on its assemblages has been conducted mostly in the western Mediterranean (Idato et al. 1983; Templado 1984; García-Raso 1990; Gambi et al. 1992, 1995; Belgacem et al. 2011; Albano and Sabelli 2012; Urra et al. 2013; Bedini et al. 2015) and in the Adriatic Sea (Solustri et al. 2002; Beqiraj et al. 2008). Molluscs in particular have never been surveyed quantitatively in *Posidonia oceanica* meadows in the eastern part of the basin, notwithstanding they are one of the most diverse taxa in the Mediterranean.

We here describe a molluscan assemblage from a *Posidonia oceanica* meadow in Plakias, south-western Crete, along a depth transect from 5 to 20 m. We sampled in spring and autumn, to capture intra-annual variation, and both the leaves and the rhizomes. The latter have often been neglected but host the richest *Posidonia oceanica* assemblages (Albano and Sabelli 2012; Albano and Stockinger 2019). We expected a poor native assemblage and diverse and abundant populations of non-indigenous species, but our results suggest the opposite, calling for stronger conservation measures of *Posidonia oceanica* meadows in this over-stressed basin.

## Materials and methods

### Study area and sampling methods

**Fig. 1:**
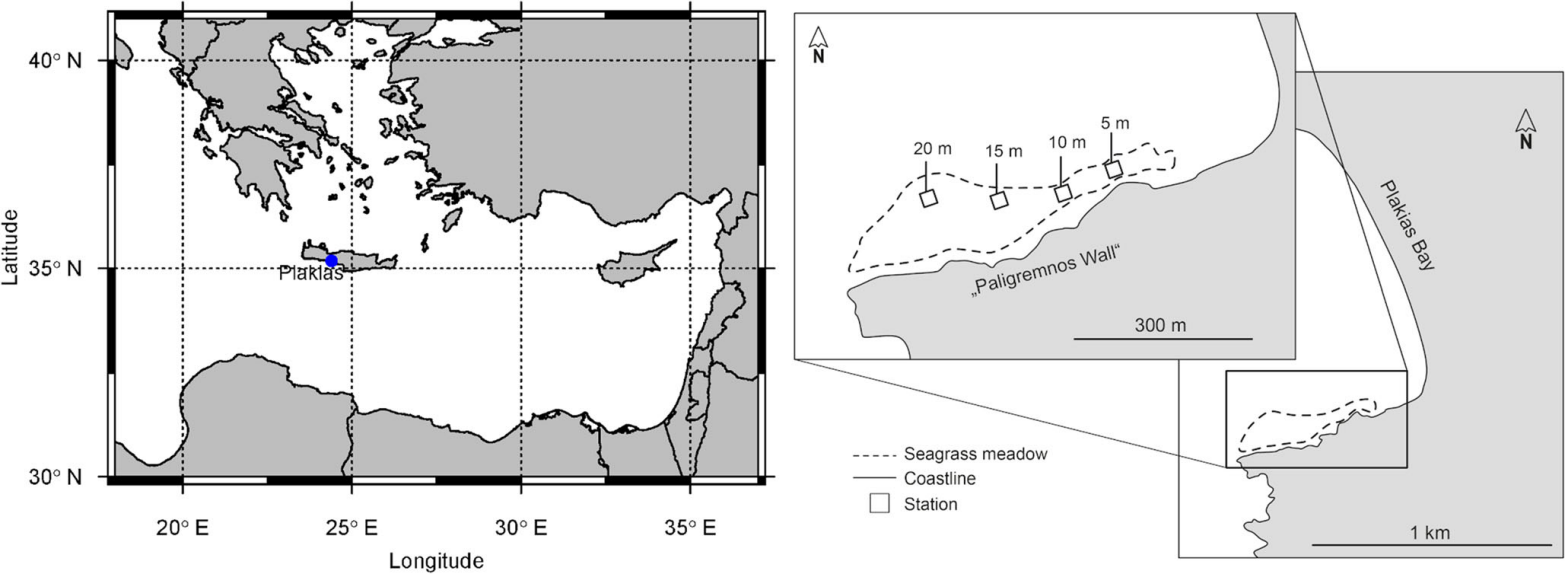
Geographic location of the study site Plakias, south-western Crete, in the eastern Mediterranean Sea (left) and detail of the sampling area in Plakias Bay (right), where the dashed line indicates the area covered by the *Posidonia oceanica* meadow. Squares mark the four different stations (5 m, 10 m, 15 m, 20 m depth)

The study was carried out in the bay of Plakias, on the south-western coast of Crete, (35.1796° N, 24.3957° E, Figure 1). The bay is 1.5 km long and extends from NNW to SSE. It is bordered on the south side by a long rock wall called “Paligremnos Wall”, which extends to the south-west into the sea for approximately 800 m. At the bottom of this wall, a sandy substrate stretches over the entire bay partly covered with a *Posidonia oceanica* meadow. The meadow is mostly found parallel to the wall, starting patchily at just 30-cm water depth, and growing into a dense meadow at a depth of 5 m continuing to reach more than 30 m. In the year of sampling, the sea surface temperature in the bay ranged from a minimum of 15 °C in March to a maximum of 27 °C in August.

**Fig. 2:**
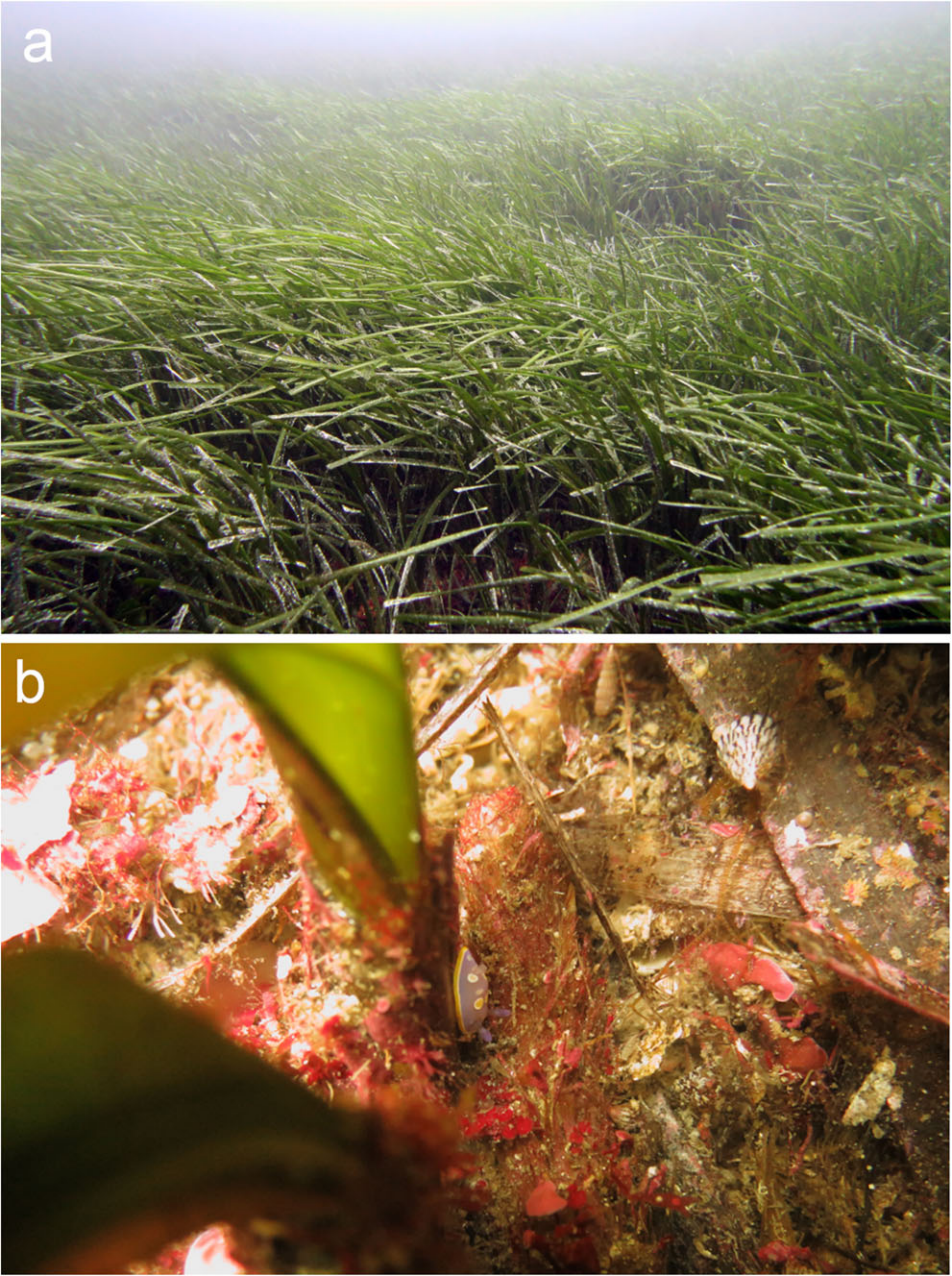
The *Posidonia oceanica* meadow at Plakias, south-western Crete. **a** The leaf stratum; **b** The rhizome stratum; note the abundance of molluscs: the vetigastropod *Jujubinus exasperatus* (top right white cone-shaped shell, grazing on a dead *Posidonia* leaf) and the nudibranch *Felimida luteorosa* (violet and yellow, in the centre of the image, identification courtesy G. Furfaro) have been captured by this randomly shot photo

Samples were taken while SCUBA diving from both the leaf and the rhizome strata (Figure 2) of the meadow at four different depths (5 m, 10 m, 15 m, 20 m) in May and in September 2017 to capture intra-annual variation. For the leaf stratum, samples were collected using a hand-operated net according to the technique described by Ledoyer (1962), modified and standardized by Russo et al. (1985). The hand net consists of a metal frame (40 × 20 cm) and has a 500-μm mesh. For each replicate, 60 strokes were given against the base of the leaves; the net is then pulled upwards to collect the mobile fauna crawling on the leaves. At every depth, we collected two and four replicates in spring and in autumn, respectively.

The rhizome stratum was sampled with an air-lift suction sampler that is 1 m long and 8 cm in diameter (Templado et al. 2010). This device consists of a PVC tube on which a net with a mesh size of 500 μm is mounted at one end. A SCUBA tank was attached to the pipe to supply air for the suction force. Sampling was carried out on three 1-m^2^ quadrats at each depth and season, after defoliation to improve sampling efficacy (Bonfitto et al. 1998). Standardization of sampling intensity was performed by using 100 bar of a 12-l steel tank for each replicate. At each 1-m^2^ replicate, the shoot density of the *Posidonia oceanica* meadow was counted on a 40 × 40 cm sub-quadrat (Panayotidis et al. 1981) and the meadow status assessed according to UNEP/MAP-RAC/SPA (2015).

Back in the lab, the samples were kept in saltwater in order to keep the organisms alive and sieved with a 1-mm mesh size. Living shelled molluscs were picked under a stereomicroscope, identified to species level and fixed in 96% ethanol.

### Data analysis

We computed sample coverage and estimated richness at perfect coverage (= 1) with the iNEXT R package (Chao and Jost 2012; Hsieh et al. 2016). We then plotted a non-metric multidimensional scaling ordination on square root transformed relative abundances to determine differences between the leaf and the rhizome strata and between the seasons. Such differences were then tested with PERMANOVA (Anderson 2001). For each station and season, we computed the number of specimens (*N*), the number of species (*S*), the Shannon diversity index (*H*’) and the Pielou’s evenness (*J*). For each species, we computed dominance (%D). The raw data arranged per replicate are available in ESM 1.

We attributed feeding guilds according to the following classification: carnivores (C) feeding on mobile organisms, such as molluscs or polychaetes; scavengers (SC) feeding on remains of dead organisms; deposit feeders (D) feeding on organic particles contained in the sediment; ectoparasites and specialized carnivores (E) feeding on much larger organisms on which they live during their life cycle; filter feeders (F) intercepting nutrient particles with their gills and/or mucous strings; macroalgae grazers (AG); seagrass grazers (SG) ingesting seagrass tissues; microalgal or periphyton grazers (MG) feeding on microalgae (e.g. diatoms); oophagus feeders (O), including species that feed on egg masses of other organisms; and symbiont-bearing species (SY) for those species in which symbiotic bacteria play an important role for obtaining a complementary energy source. This is the same classification of feeding modes and guilds used by Rueda et al. (2009) and Albano and Sabelli (2012) for comparative purposes. Trophic information for all species was obtained from the literature.

We determined the geographic range, with particular focus on endemic and non-indigenous species, from the literature. Molluscan species authorities are reported in the Tables 3 and 6 displaying abundance data; therefore, we do not mention them in the text. The systematic arrangement follows Sigwart et al. (2013) for the polyplacophorans, Bouchet et al. (2017) for the gastropods, Bouchet et al. (2010) for the bivalves, and Steiner and Kabat (2001) for the scaphopods. All data analyses and plotting were carried out in the R statistical environment (R Development Core Team 2019).

## Results

### *Posidonia oceanica* bed structure

The shoot density of *Posidonia oceanica* in the meadow decreases with depth. The highest shoot density of 740 ± 185 shoots m^−2^ was at 5-m depth. Density decreased to a minimum of 490 ± 52 shoots m^−2^ at 20-m depth (Table 1). Still, the status of the meadow increases with depth from good to high, because the decrease of shoot density with depth is naturally associated with decreased sun irradiation (Pergent et al. 1995). This trend may suggest that the shallowest sampling stations experience some degree of anthropic disturbance, in contrast to undisturbed deeper ones.

**Table 1:** Shoot density (shoots m^−2^) of the *Posidonia oceanica* meadow at Plakias, south-western Crete, with the final status classification according to UNEP/MAP-RAC/SPA (2015)

Replicate	Shoot density (shoots m−^2^)			
	5-m depth	10-m depth	15-m depth	20-m depth
1	487	631	681	506
2	975	662	587	537
3	681	631	681	493
4	693	706	581	431
5	943	612	500	550
6	662	687	593	424
Mean ± SD (status)	740 ± 185 (Good)	655 ± 36 (Good)	604 ± 68 (High)	490 ± 52 (High)

### The molluscan taxocoenosis

**Fig. 3:**
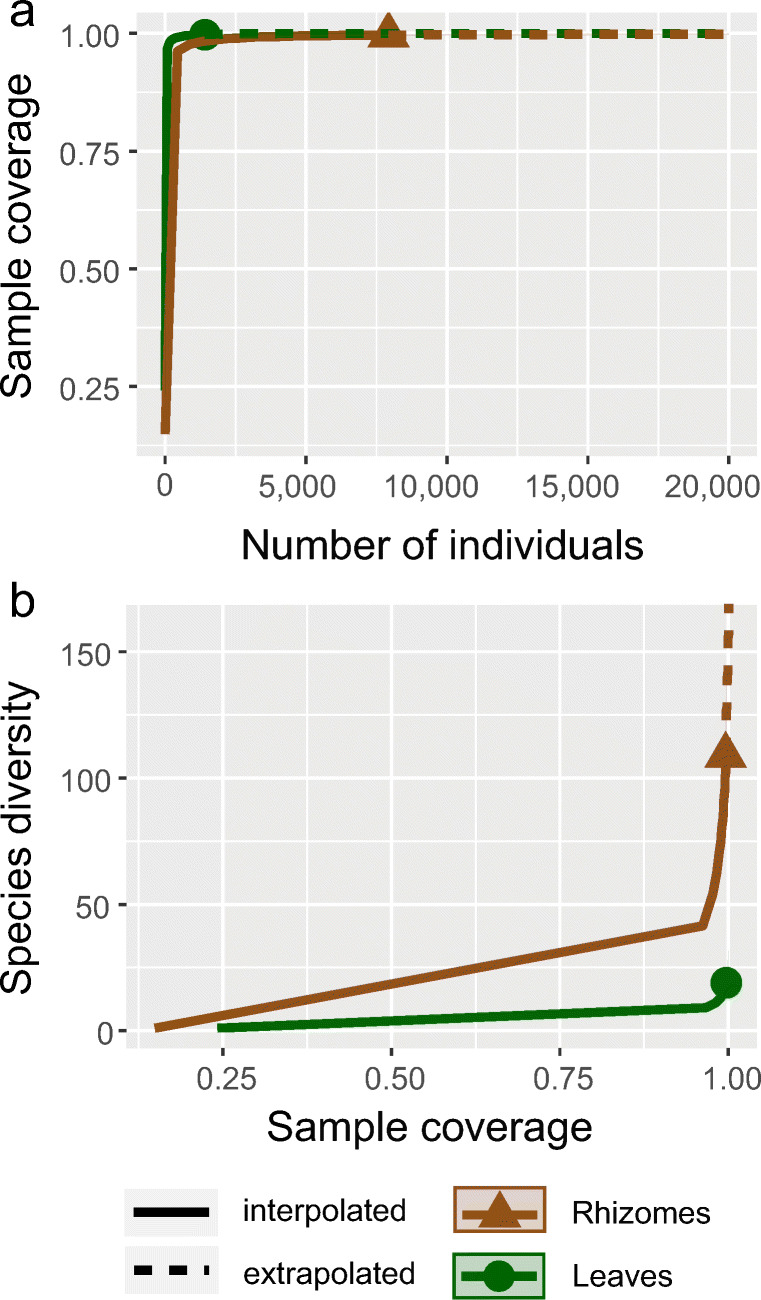
Sample coverage and estimated species richness at perfect coverage (= 1) of the leaf and rhizome strata of the *Posidonia oceanica* meadow in Plakias, south-western Crete. **a** Sample coverage reaches 99.7% and 99.6% in the leaf and rhizome stratum, respectively; **b** Notwithstanding the high coverage, the extrapolated diversity at perfect coverage is 23 (+21%) and 169 (+55%) species on the leaf and rhizome stratum, respectively

We collected 9344 specimens, belonging to 109 species: 75 (68.8%) gastropods, 30 (27.5%) bivalves and 4 (3.7%) polyplacophorans. Species richness was 19 and 108 species in the leaf and rhizome stratum, respectively, with a very high sample coverage: 99.7% and 99.6%, respectively. Still, the extrapolated diversity at perfect coverage (= 1) was 23 (+21%) and 169 (+55%) species, respectively (Figure 3).

**Fig. 4:**
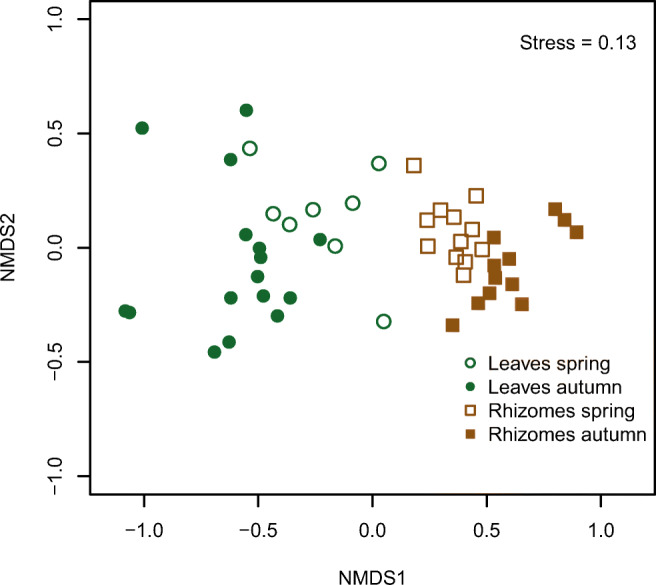
Non-metric multidimensional scaling plot of molluscan assemblages in the leaf and rhizome strata of the *Posidonia oceanica* meadow in Plakias, south-western Crete. The molluscan assemblages differ between the strata and the seasons

**Fig. 5:**
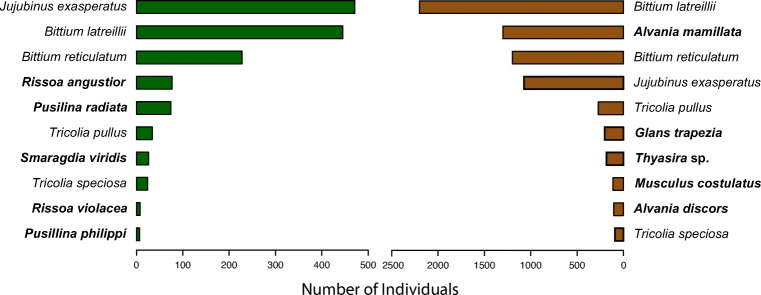
Comparison of the ten most abundant species in the leaf (left, green) and the rhizome stratum (right, brown) of the *Posidonia oceanica* meadow in south-western Crete. The species in bold are those present in only one of the strata among the ten most abundant here illustrated

The non-metric multidimensional scaling plot shows that the molluscan assemblages of the leaf and rhizome strata were segregated into two distinct clouds of points (Figure 4). Indeed, these assemblages were significantly different (PERMANOVA, Fd = 29.83, *R*^2^=0.39, *p*=0.001) suggesting that they can be treated as different entities in the following paragraphs. Such differences were reflected in the most abundant species assemblage composition: in each stratum, five of the ten most abundant species were not equally abundant in the other stratum (Figure 5). Additionally, the rhizome stratum hosted most of the sampled individuals (7930 vs 1414 in the leaf stratum) and species (108 vs 19 in the leaf stratum). The differences between the seasons were very clear for the rhizomes and less neat for the leaves, but still statistically significant in both cases (PERMANOVA, leaf stratum: Fd = 5.18, *R*^2^ = 0.19, *p* = 0.001; rhizome stratum: Fd = 6.23, *R*^2^ = 0.22, *p* = 0.001).

The assemblage contained 35 (0.3%) individuals of five species endemic to the eastern Mediterranean (*Alvania bozcaadensis*, *Aegeofusinus rolani*, *Ocinebrina aegeensis*, *Chlathromangelia loiselieri* and *Parvicardium trapezium*) and 98 (1.0%) individuals of four non-indigenous species (*Viriola* cf. *bayani*, *Septifer cumingii*, *Isognomon* aff. *australica* and *Pinctada radiata*), all of Indo-Pacific origin.

### The molluscan assemblage on the leaves

In the leaf stratum, we collected 1414 individuals belonging to 19 species. Sampling saturation was excellent, exceeding a 98% coverage at all sampling events; still, the observed richness at 5-m depth in autumn and at 20-m depth in spring was largely underestimated (Table 2). Nearly all collected individuals are gastropods, except two individuals of bivalves, one belonging to the only non-indigenous species found among the leaves (*Pinctada radiata* in 20 m depth). The most abundant species were *Jujubinus exasperatus*, *Bittium latreillii* and *Bittium reticulatum*, represented by more than a hundred specimens each. The differences between the spring and autumn assemblages are mostly driven by different proportions of these species that are more dominant in spring. *Rissoa violacea* was the only species found exclusively in the leaf stratum. A detailed list of species per depth, season and their dominance, is given in Table 3. At the shallowest sites, Shannon index and Pielou’s evenness are markedly lower in autumn than in spring (Table 2). In terms of feeding guilds, the molluscan assemblage of the leaves is dominated by microalgal grazers with a minor component of seagrass-feeding herbivores, filter feeders and carnivores (Table 4).

**Table 2:** Diversity indices of the molluscan assemblage on the leaves of the *Posidonia oceanica* meadow in Plakias, south-western Crete: *N* abundance, *S*_*obs*_ observed species richness, *S*_*est*_ estimated species richness at perfect coverage, coverage sensu Chao and Jost (2012), *H’* Shannon index, *J* Pielou’s evenness

Depth	*N*		*S*_obs_		*S*_est_		Coverage		*H*’		*J*	
	Spring	Autumn	Spring	Autumn	Spring	Autumn	Spring	Autumn	Spring	Autumn	Spring	Autumn
5 m	288	164	8	10	9 (+12.5%)	16 (+60%)	0.996	0.976	1.507	1.294	0.725	0.562
10 m	183	131	10	8	11 (+10%)	9 (+12.5%)	0.989	0.985	1.586	0.945	0.689	0.454
15 m	61	33	8	8	8 (+0%)	8 (+0%)	0.983	0.977	1.488	1.667	0.716	0.802
20 m	424	130	11	12	17 (+54.5%)	14 (+16.7%)	0.991	0.985	1.179	1.714	0.492	0.690

**Table 3:** Quali-quantitative list of the molluscs found in the leaf stratum of the *Posidonia oceanica* meadow in Plakias, south-western Crete. For each species, we report the feeding guild and the dominance (between brackets). The feeding guild codes are: *C* carnivores feeding on mobile organisms, *SC* scavengers, *D* deposit feeders, *E* ectoparasites and specialized carnivores, *F* filter feeders, *AG* macroalgae grazers, *SG* seagrass grazers, *MG* microalgal or periphyton grazers, *O* oophagus feeders, *SY* symbiont-bearing species. Species endemic to the eastern Mediterranean are marked with an asterisk. Non-indigenous species are marked in bold

	Feeding guild	5-m depth		10-m depth		15-m depth		20-m depth	
		Spring	Autumn	Spring	Autumn	Spring	Autumn	Spring	Autumn
*Jujubinus exasperatus* (Pennant, 1777)	MG	59 (20.5%)	85 (51.8%)	95 (51.9%)	98 (74.8%)	31 (55.4%)	16 (42.1%)	31 (7.3%)	56 (43.1%)
*Calliostoma laugieri* (Payraudeau, 1826)	MG	2 (0.7%)	1 (0.6%)	-	1 (0.8%)	-	-	-	-
*Tricolia speciosa* (Megerle von Mühlfeld, 1824)	MG	9 (3.1%)	3 (1.8%)	1 (0.6%)	1 (0.8%)	2 (3.6%)	2 (5.3%)	3 (0.7%)	3 (2.3%)
*Tricolia pullus* (Linnaeus, 1758)	MG	-	11 (6.7%)	6 (3.3%)	8 (6.1%)	2 (3.6%)	-	-	7 (5.4%)
*Smaragdia viridis* (Linnaeus, 1758)	SG	5 (1.7%)	1 (0.6%)	7 (3.8%)	-	6 (10.7%)	2 (5.3%)	5 (1.2%)	-
*Bittium latreillii* (Payraudeau, 1826)	MG	105 (36.5%)	51 (31.1%)	9 (4.9%)	15 (11.5%)	6 (10.7%)	3 (7.9%)	248 (58.5%)	8 (6.2%)
*Bittium reticulatum* (da Costa, 1778)	MG	79 (27.4%)	4 (2.4%)	28 (15.3%)	2 (1.5%)	-	-	112 (26.4%)	3 (2.3%)
*Alvania mamillata* Risso, 1826	MG	-	-	-	-	-	-	1 (0.2%)	1 (0.8%)
*Pusillina philippi* (Aradas & Maggiore, 1844)	MG	-	1 (0.6%)	-	-	-	-	-	6 (4.6%)
*Pusillina radiata* (Philippi, 1836)	MG	1 (0.4%)	-	16 (8.7%)	2 (1.5%)	3 (5.4%)	2 (5.3%)	15 (3.5%)	35 (26.9%)
*Alvania lineata* Risso, 1826	MG	-	1 (0.6%)	-	-	-	-	-	1 (0.8%)
*Rissoa angustior* (Monterosato, 1917)	MG	28 (9.7%)	6 (3.7%)	15 (8.2%)	4 (3.1%)	5 (8.9%)	9 (23.7%)	6 (1.4%)	4 (3.1%)
*Rissoa violacea* Desmarest, 1814	MG	-	-	-	-	-	3 (7.9%)	1 (0.2%)	4 (3.1%)
*Granulina marginata* (Bivona, 1832)	C	-	-	5 (2.7%)	-	-	-	-	-
*Muricopsis cristata* (Brocchi, 1814)	C	-	-	-	-	1 (1.8%)	-	-	-
*Ocinebrina aegeensis Aissaoui, Barco & Oliverio, 2017	C	-	-	1 (0.6%)	-	-	-	-	2 (1.5%)
*Aplysia parvula* Mörch, 1863	MG	-	-	-	-	-	-	1 (0.2%)	-
*Barbatia barbata* (Linnaeus, 1758)	F	-	-	-	-	-	1 (2.6%)	-	-
***Pinctada radiata*** **(Leach, 1814)**	**F**	**-**	**-**	**-**	**-**	**-**	**-**	**1 (0.2%)**	**-**

**Table 4:** Feeding guild structure of the molluscan assemblage on the leaves of the *Posidonia oceanica* meadow in Plakias, south-western Crete

		5-m depth		10-m depth		15-m depth		20-m depth	
		Spring	Autumn	Spring	Autumn	Spring	Autumn	Spring	Autumn
MG, microalgae herbivores	Specimens	98.3%	98.8%	92.9%	100%	87.5%	92.1%	98.6%	98.5%
	Species	87.5%	90.0%	70.0%	100%	75.0%	75.0%	81.8%	91.7%
SG, seagrass-feeding herbivores	Specimens	1.7%	0.6%	3.8%	-	10.7%	5.3%	1.2%	-
	Species	12.5%	10.0%	10.0%	-	12.5%	12.5%	9.1%	-
F, filter feeders	Specimens	-	-	-	-	-	2.6%	0.2%	-
	Species	-	-	-	-	-	12.5%	9.1%	-
C, carnivores on mobile prey	Specimens	-	-	3.3%	-	1.8%		-	1.5%
	Species	-	-	20.0%	-	12.5%	-	-	8.3%
Carnivorous/microalgae herbivores ratio	Specimens	-	-	0.03	-	0.02	-	-	0.02

### The molluscan assemblage in the rhizomes

In the rhizome stratum, we collected 7930 individuals belonging to 108 species: 74 (68.5%) gastropods, 30 (27.8 %) bivalves and 4 (3.7%) polyplacophorans. Sampling saturation was excellent, exceeding a 98% coverage at all sampling events; still, the observed richness was largely underestimated, with an estimated richness ~16.7–192.6% larger than the observed one (Table 5). The most abundant species were *Bittium latreillii*, *Alvania mamillata*, *Bittium reticulatum* and *Jujubinus exasperatus*, with more than a thousand individuals each. The differences between the spring and autumn assemblages are mostly driven by different proportions of these species that are more dominant in spring. A detailed list, of species per depth, season and their dominance, is given in Table 6. Shannon diversity was much higher than in the leaf stratum whereas both Shannon diversity and Pielou’s evenness values were more stable between seasons (Table 5). The trophic structure of the molluscan assemblage in the rhizomes was much more diverse than in the leaves: eight guilds out of ten were represented (Table 7). Only scavengers and oophagus feeders were missing. More than 80% of the assemblage abundance is composed by microalgal grazers, with a non-marginal component of filter feeders.

**Table 5:** Diversity indices of the molluscan assemblage in the rhizomes of a *Posidonia oceanica* meadow in Plakias, south-western Crete: *N* abundance, *S*_*obs*_ observed species richness, *S*_*est*_ estimated species richness at perfect coverage, coverage sensu Chao and Jost (2012), *H’* Shannon index, *J* Pielou’s evenness

Depth	*N*		*S*_obs_		*S*_est_		Coverage		*H*’		*J*	
	Spring	Autumn	Spring	Autumn	Spring	Autumn	Spring	Autumn	Spring	Autumn	Spring	Autumn
5 m	423	1000	30	63	47 (+56.7%)	98 (+55.6%)	0.976	0.978	2.387	2.960	0.702	0.714
10 m	1100	1058	48	50	56 (+16.7%)	71 (+42.0%)	0.987	0.984	2.271	2.283	0.587	0.584
15 m	743	1309	38	51	66 (+73.7%)	70 (+37.3%)	0.980	0.989	2.197	2.175	0.604	0.553
20 m	1458	839	54	46	158 (+192.6%)	69 (+50.0%)	0.983	0.982	2.195	2.234	0.550	0.583

**Table 6:** Quali-quantitative list of the molluscs found in the rhizome stratum of the *Posidonia oceanica* meadow in Plakias, south-western Crete. For each species, we report the feeding guild and the dominance (between brackets). The feeding guild codes are: *C* carnivores feeding on mobile organisms, *SC* scavengers, *D* deposit feeders, *E* ectoparasites and specialized carnivores, *F* filter feeders, *AG* macroalgae grazers, *SG* seagrass grazers, *MG* microalgal or periphyton grazers, *O* oophagus feeders, *SY* symbiont-bearing species. Species endemic to the eastern Mediterranean are marked with an asterisk. Non-indigenous species are marked in bold

	Feeding guild	5-m depth	5-m depth	10-m depth	10-m depth	15-m depth	15-m depth	20-m depth	20-m depth
		Spring	Autumn	Spring	Autumn	Spring	Autumn	Spring	Autumn
*Acanthochitona fascicularis* (Linnaeus, 1767)	MG	-	2 (0.2%)	1 (0.1%)	5 (0.5%)	2 (0.3%)	3 (0.2%)	8 (0.5%)	10 (1.2%)
*Leptochiton bedullii* Dell'Angelo & Palazzi, 1986	MG	-	4 (0.4%)	-	2 (0.2%)	-	4 (0.3%)	7 (0.5%)	-
*Callochiton septemvalvis* (Montagu, 1803)	MG	-	1 (0.1%)	-	-	-	2 (0.2%)	1 (0.1%)	-
*Rhyssoplax olivacea* (Spengler, 1797)	MG	-	-	-	-	1 (0.1%)	1 (0.1%)	1 (0.1%)	-
*Patella* sp.	MG	-	-	-	-	1 (0.1%)	-	-	-
*Emarginula sicula* J.E. Gray, 1825	E	-	-	1 (0.1%)	1 (0.1%)	-	1 (0.1%)	-	-
*Scissurella costata* d'Orbigny, 1824	MG	8 (1.9%)	5 (0.5%)	26 (2.4%)	-	16 (2.2%)	3 (0.2%)	17 (1.2%)	-
*Clanculus cruciatus* (Linnaeus, 1758)	MG	-	-	-	-	-	-	1 (0.1%)	-
*Gibbula ardens* (Salis Marschlins, 1793)	MG	-	-	1 (0.1%)	-	-	-	-	-
*Jujubinus exasperatus* (Pennant, 1777)	MG	40 (9.5%)	47 (4.7%)	184 (16.7%)	113 (10.7%)	99 (13.3%)	200 (15.3%)	287 (19.7%)	105 (12.5%)
*Jujubinus striatus* (Linnaeus, 1758)	MG	-	-	2 (0.2%)	1 (0.1%)	-	3 (0.2%)	-	2 (0.2%)
*Steromphala varia* (Linnaeus, 1758)	MG	-	1 (0.1%)	-	-	-	-	-	-
*Calliostoma laugieri* (Payraudeau, 1826)	MG	1 (0.2%)	24 (2.4%)	2 (0.2%)	2 (0.2%)	1 (0.1%)	-	-	-
*Homalopoma sanguineum* (Linnaeus, 1758)	MG	-	-	-	-	-	-	-	1 (0.1%)
*Tricolia pullus* (Linnaeus, 1758)	MG	40 (9.5%)	69 (6.9%)	42 (3.8%)	24 (2.3%)	28 (3.8%)	21 (1.6%)	27 (1.9%)	21 (2.5%)
*Tricolia speciosa* (Megerle von Mühlfeld, 1824)	MG	3 (0.7%)	8 (0.8%)	31 (2.8%)	8 (0.8%)	12 (1.6%)	12 (0.9%)	14 (1%)	5 (0.6%)
*Tricolia tenuis* (Michaud, 1829)	MG	-	4 (0.4%)	-	-	-	-	-	-
*Bolma rugosa* (Linnaeus, 1767)	MG	-	1 (0.1%)	-	2 (0.2%)	-	1 (0.1%)	1 (0.1%)	6 (0.7%)
*Smaragdia viridis* (Linnaeus, 1758)	SG	14 (3.3%)	27 (2.7%)	14 (1.3%)	9 (0.9%)	5 (0.7%)	12 (0.9%)	3 (0.2%)	8 (1%)
*Bittium latreillii* (Payraudeau, 1826)	MG	99 (23.4%)	142 (14.2%)	190 (17.3%)	269 (25.4%)	214 (28.8%)	471 (36%)	464 (31.8%)	353 (42.1%)
*Bittium reticulatum* (da Costa, 1778)	MG	103 (24.3%)	42 (4.2%)	317 (28.8%)	119 (11.2%)	169 (22.7%)	96 (7.3%)	291 (20%)	60 (7.2%)
*Cerithium* sp.	MG	-	1 (0.1%)	-	-	-	-	1 (0.1%)	1 (0.1%)
*Turritella turbona* Monterosato, 1877	F	-	-	-	-	-	2 (0.2%)	-	-
*Notocochlis dillwynii* (Payraudeau, 1826)	C	1 (0.2%)	-	1 (0.1%)	-	1 (0.1%)	-	2 (0.1%)	-
*Marshallora adversa* (Montagu, 1803)	E	-	1 (0.1%)	1 (0.1%)	-	-	1 (0.1%)	1 (0.1%)	-
*Monophorus erythrosoma* (Bouchet & Guillemot, 1978)	E	-	-	-	-	-	-	1 (0.1%)	-
*Monophorus perversus* (Linnaeus, 1758)	E	-	1 (0.1%)	-	-	-	-	-	-
*Similiphora similior* (Bouchet & Guillemot, 1978)	E	-	1 (0.1%)	1 (0.1%)	-	1 (0.1%)	-	-	-
***Viriola*** **cf.** ***bayani*** **Jousseaume, 1884**	**E**	**-**	**-**	**-**	**1 (0.1%)**	**-**	**-**	**-**	**1 (0.1%)**
*Cerithiopsis tubercularis* (Montagu, 1803)	E	-	4 (0.4%)	-	-	1 (0.1%)	-	-	-
*Alvania bozcaadensis Tisselli & Giunchi, 2013	MG	-	-	-	-	-	-	-	3 (0.4%)
*Alvania clarae* Nofroni & Pizzini, 1991	MG	-	2 (0.2%)	-	-	-	-	-	-
*Alvania discors* (Allan, 1818)	MG	14 (3.3%)	57 (5.7%)	8 (0.7%)	2 (0.2%)	-	-	15 (1%)	10 (1.2%)
*Alvania lineata* Risso, 1826	MG	5 (1.2%)	7 (0.7%)	-	-	-	-	-	-
*Alvania mamillata* Risso, 1826	MG	29 (6.9%)	196 (19.6%)	152 (13.8%)	306 (28.9%)	104 (14%)	279 (21.3%)	119 (8.2%)	115 (13.7%)
*Pusillina philippi* (Aradas & Maggiore, 1844)	MG	-	-	-	1 (0.1%)	1 (0.1%)	-	1 (0.1%)	-
*Pusillina radiata* (Philippi, 1836)	MG	-	1 (0.1%)	10 (0.9%)	3 (0.3%)	5 (0.7%)	11 (0.8%)	8 (0.5%)	11 (1.3%)
*Rissoa angustior* (Monterosato, 1917)	MG	20 (4.7%)	6 (0.6%)	3 (0.3%)	4 (0.4%)	2 (0.3%)	1 (0.1%)	1 (0.1%)	3 (0.4%)
*Rissoina bruguieri* (Payraudeau, 1826)	MG	-	3 (0.3%)	-	-	-	-	-	-
*Caecum auriculatum* De Folin, 1868	MG	-	1 (0.1%)	-	-	-	-	1 (0.1%)	1 (0.1%)
*Caecum clarkii* Carpenter, 1859	MG	-	-	-	-	-	1 (0.1%)	-	-
Eulimidae sp. 1	E	-	2 (0.2%)	2 (0.2%)	-	-	-	-	-
*Melanella lubrica* (Monterosato, 1890)	E	-	2 (0.2%)	-	-	-	-	-	1 (0.1%)
*Vitreolina philippi* (de Rayneval & Ponzi, 1854)	E	-	2 (0.2%)	-	-	-	-	-	-
*Lamellaria perspicua* (Linnaeus, 1758)	E	-	-	-	-	-	-	1 (0.1%)	-
*Gibberula philippii* (Monterosato, 1878)	C	-	-	-	-	-	5 (0.4%)	-	-
*Granulina marginata* (Bivona, 1832)	C	-	-	2 (0.2%)	11 (1%)	-	1 (0.1%)	-	-
*Volvarina mitrella* (Risso, 1826)	C	-	1 (0.1%)	-	-	-	-	-	-
*Chauvetia turritellata* (Deshayes, 1835)	C	-	2 (0.2%)	2 (0.2%)	3 (0.3%)	-	-	-	-
*Euthria cornea* (Linnaeus, 1758)	C	-	2 (0.2%)	-	1 (0.1%)	-	-	-	-
*Aegeofusinus rolani (Buzzurro & Ovalis, 2005)	C	1 (0.2%)	3 (0.3%)	-	1 (0.1%)	-	2 (0.2%)	-	2 (0.2%)
*Aptyxis syracusana* (Linnaeus, 1758)	C	-	1 (0.1%)	-	-	-	-	-	-
*Hexaplex trunculus* (Linnaeus, 1758)	C	-	7 (0.7%)	2 (0.2%)	1 (0.1%)	1 (0.1%)	1 (0.1%)	1 (0.1%)	2 (0.2%)
*Muricopsis cristata* (Brocchi, 1814)	C	1 (0.2%)	4 (0.4%)	3 (0.3%)	10 (0.9%)	1 (0.1%)	12 (0.9%)	1 (0.1%)	2 (0.2%)
*Ocinebrina aegeensis Aissaoui, Barco & Oliverio, 2017	C	-	-	1 (0.1%)	2 (0.2%)	-	4 (0.3%)	1 (0.1%)	-
*Typhinellus labiatus* (de Cristofori & Jan, 1832)	C	-	-	2 (0.2%)	1 (0.1%)	1 (0.1%)	-	-	1 (0.1%)
*Haedropleura* aff. *secalina* (Philippi, 1844)	C	-	-	-	3 (0.3%)	-	6 (0.46%)	1 (0.1%)	1 (0.1%)
*Mangelia* sp.	C	-	-	1 (0.1%)	-	-	-	-	-
*Clathromangelia granum* (Philippi, 1844)	C	-	1 (0.1%)	-	-	-	2 (0.2%)	-	1 (0.1%)
*Clathromangelia loiselieri Oberling, 1970	C	-	-	-	2 (0.2%)	-	4 (0.3%)	2 (0.1%)	4 (0.5%)
*Raphitoma* sp.	C	-	-	-	1 (0.1%)	-	-	-	-
*Retusa truncatula* (Bruguière, 1792)	C	5 (1.2%)	-	11 (1%)	-	5 (0.7%)	-	8 (0.5%)	-
*Weinkauffia macandrewii* (E. A. Smith, 1872)	MG	-	-	-	-	1 (0.1%)	-	-	-
*Philine catena* (Montagu, 1803)	C	2 (0.5%)	-	1 (0.1%)	-	-	-	1 (0.1%)	-
*Aplysia depilans* Gmelin, 1791	MG	-	-	-	-	-	-	1 (0.1%)	-
*Aplysia parvula* Mörch, 1863	MG	-	-	1 (0.1%)	-	-	-	3 (0.2%)	-
*Ascobulla fragilis* (Jeffreys, 1856)	AG	-	-	-	1 (0.1%)	-	-	-	-
*Williamia gussoni* (Costa O.G., 1829)	AG	3 (0.7%)	-	4 (0.4%)	-	6 (0.8%)	-	14 (1%)	-
*Eulimella acicula* (Philippi, 1836)	E	-	-	-	-	-	-	1 (0.1%)	-
*Odostomia acuta* Jeffreys, 1848	E	-	-	1 (0.1%)	1 (0.1%)	-	-	-	-
*Megastomia conoidea (Brocchi, 1814)*	E	-	1 (0.1%)	2 (0.2%)	1 (0.1%)	-	-	-	-
*Odostomia turriculata* Monterosato, 1869	E	-	-	-	-	-	-	1 (0.1%)	-
*Ondina vitrea* (Brusina, 1866)	E	-	-	-	5 (0.5%)	-	1 (0.1%)	-	-
*Parthenina interstincta* (J. Adams, 1797)	E	-	1 (0.1%)	1 (0.1%)	-	-	-	-	-
*Parthenina monterosatii* (Clessin, 1900)	E	-	1 (0.1%)	-	-	-	-	-	-
*Parthenina terebellum* (Philippi, 1844)	E	-	1 (0.1%)	-	-	-	-	-	-
*Pyrgostylus striatulus* (Linnaeus, 1758)	E	-	5 (0.5%)	4 (0.4%)	6 (0.6%)	-	1 (0.1%)	-	8 (1%)
*Notodiaphana atlantica* Ortea, Moro & Espinosa, 2013	NA	-	1 (0.1%)	-	-	-	-	-	-
*Nucula nitidosa* Winckworth, 1930	D	-	20 (2%)	2 (0.2%)	3 (0.3%)	1 (0.1%)	5 (0.4%)	7 (0.5%)	3 (0.4%)
*Crenella arenaria* Monterosato, 1875 ex H. Martin, ms.	F	1 (0.2%)	16 (1.6%)	-	3 (0.3%)	2 (0.3%)	9 (0.7%)	17 (1.2%)	6 (0.7%)
*Gregariella semigranata* (Reeve, 1858)	F	-	-	-	1 (0.1%)	-	1 (0.1%)	1 (0.1%)	-
*Modiolula phaseolina* (Philippi, 1844)	F	-	-	-	1 (0.1%)	-	-	-	-
*Musculus costulatus* (Risso, 1826)	F	11 (2.6%)	59 (5.9%)	7 (0.6%)	6 (0.6%)	15 (2%)	10 (0.8%)	5 (0.3%)	1 (0.1%)
***Septifer cumingii*** **Récluz, 1848**	F	**6 (1.4%)**	**18 (1.8%)**	**-**	**8 (0.8%)**	**6 (0.8%)**	**7 (0.5%)**	**3 (0.2%)**	**3 (0.4%)**
*Barbatia barbata* (Linnaeus, 1758)	F	3 (0.7%)	12 (1.2%)	3 (0.3%)	4 (0.4%)	4 (0.5%)	7 (0.5%)	2 (0.1%)	4 (0.5%)
*Arca noae* Linnaeus, 1758	F	1 (0.2%)	4 (0.4%)	2 (0.2%)	-	1 (0.1%)	-	1 (0.1%)	1 (0.1%)
*Striarca lactea* (Linnaeus, 1758)	F	4 (0.9%)	7 (0.7%)	7 (0.6%)	16 (1.5%)	8 (1.1%)	14 (1.1%)	10 (0.7%)	12 (1.4%)
***Isognomon*** **aff. australica (Reeve, 1858)**	F	**-**	**1 (0.1%)**	**-**	**-**	**-**	**-**	**-**	**-**
***Pinctada radiata*** **(Leach, 1814)**	F	**2 (0,5%)**	**8 (0.8%)**	**4 (0.4%)**	**1 (0.1%)**	**9 (1.2%)**	**1 (0.1%)**	**12 (0.8%)**	**6 (0.7%)**
*Pinna nobilis* Linnaeus, 1758	F	1 (0.2%)	-	2 (0.2%)	-	3 (0.4%)	-	1 (0.1%)	-
*Flexopecten hyalinus* (Poli, 1795)	F	1 (0.2%)	5 (0.5%)	2 (0.2%)	-	-	4 (0.3%)	3 (0.2%)	3 (0.4%)
*Lima* (Linnaeus, 1758)	F	-	-	1 (0.1%)	-	-	2 (0.2%)	1 (0.1%)	1 (0.1%)
*Limatula subauriculata* (Montagu, 1808)	F	-	13 (1.3%)	2 (0.2%)	6 (0.6%)	2 (0.3%)	25 (1.9%)	21 (1.4%)	7 (0.8%)
*Ctena decussata* (O.G. Costa, 1829)	SY	-	7 (0.7%)	-	11 (1%)	-	6 (0.5)	8 (0.5%)	11 (1.3%)
*Loripinus fragilis* (Philippi, 1836)	SY	-	-	-	-	-	-	-	1 (0.1%)
*Megaxinus unguiculinus* Pallary, 1904	SY	-	-	-	-	-	-	-	1 (0.1%)
*Myrtea spinifera* (Montagu, 1803)	SY	-	-	-	-	-	1 (0.1%)	-	-
*Thyasira* sp.	SY	1 (0.2%)	25 (2.5%)	37 (3.4%)	34 (3.2%)	7 (0.9%)	25 (1.9%)	40 (2.7%)	14 (1.7%)
*Cardita calyculata* (Linnaeus, 1758)	F	-	-	-	1 (0.1%)	-	3 (0.3%)	-	-
*Cardites antiquatus* (Linnaeus, 1758)	F	-	-	-	-	-	7 (0.5%)	1 (0.1%)	1 (0.1%)
*Glans trapezia* (Linnaeus, 1767)	F	2 (0.5%)	102 (10.2%)	3 (0.3%)	38 (3.6%)	5 (0.7%)	14 (1.1%)	16 (1.1%)	23 (2.7%)
*Parvicardium trapezium Cecalupo & Quadri, 1996	F	-	4 (0.4%)	-	-	1 (0.1%)	-	-	-
*Arcopella balaustina* (Linnaeus, 1758)	D	-	1 (0.1%)	-	1 (0.1%)	-	2 (0.2%)	1 (0.1%)	1 (0.1%)
*Donax semistriatus* Poli, 1795	F	1 (0.2%)	-	-	-	-	-	-	-
*Abra alba* (W. Wood, 1802)	D	-	1 (0.1%)	-	2 (0.2%)	-	-	-	-
*Gouldia minima* (Montagu, 1803)	F	-	-	1 (0.1%)	-	1 (0.1%)	1 (0.1%)	-	2 (0.2%)
*Lajonkairia lajonkairii* (Payraudeau, 1826)	F	-	1 (0.1%)	-	-	-	1 (0.1%)	-	-
*Hiatella arctica* (Linnaeus, 1767)	F	-	1 (0.1%)	-	-	-	-	-	-

**Table 7:** Feeding guild structure of the molluscan assemblage on the rhizomes of the *Posidonia oceanica* meadow in Plakias, south-western Crete

		5-m depth		10-m depth		15-m depth		20-m depth	
		Spring	Autumn	Spring	Autumn	Spring	Autumn	Spring	Autumn
AG, herbivores of macroalgae and epiphytes	Specimens	0.7%	-	0.4%	0.1%	0.8%	-	1.0%	-
	Species	3.3%	-	2.1%	2.0%	2.6%	-	1.9%	-
MG, microalgae herbivores	Specimens	85.6%	62.4%	88.2%	81.4%	88.3%	84.7%	87.0%	84.3%
	Species	36.7%	34.9%	31.3%	30.0%	39.5%	31.4%	38.9%	34.8%
SG, seagrass-feeding herbivores	Specimens	3.3%	2.7%	1.3%	0.9%	0.7%	0.9%	0.2%	1.0%
	Species	3.3%	1.6%	2.1%	2.0%	2.6%	2.0%	1.9%	2.2%
D, deposit feeders	Specimens	-	2.2%	0.2%	0.6%	0.1%	0.5%	0.5%	0.5%
	Species	-	4.8%	2.1%	6.0%	2.6%	3.9%	3.7%	4.3%
F, filter feeders	Specimens	7.8%	25.1%	3.1%	8.0%	7.7%	8.3%	6.4%	8.3%
	Species	36.7%	22.2%	22.9%	22.0%	31.6%	31.4%	25.9%	28.3%
SY, symbiont-bearing	Specimens	0.2%	3.2%	3.4%	4.3%	0.9%	2.4%	3.3%	3.2%
	Species	0.9%	3.2%	2.1%	4.0%	2.6%	5.9%	3.7%	8.7%
E, ectoparasites and carnivores on preys without mobility	Specimens	-	2.2%	1.2%	1.4%	0.3%	0.3%	0.3%	1.2%
	Species	-	19.0%	16.7%	12.0%	5.3%	7.8%	9.3%	6.5%
C, carnivores on mobile prey	Specimens	2.4%	2.1%	2.4%	3.4%	1.2%	2.8%	1.2%	1.5%
	Species	16.7%	12.7%	20.8%	22.0%	13.2%	17.6%	14.8%	15.2%
Carnivorous/microalgae herbivores ratio	Specimens	0.02	0.04	0.04	0.05	0.02	0.03	0.02	0.03

## Discussion

### A diverse native molluscan assemblage, devoid of non-indigenous species

The molluscan assemblage of the *Posidonia oceanica* meadow in Plakias was abundant and diverse and hosted a negligible non-indigenous component. A direct comparison with the few previous works which inspected both the leaf and rhizome strata and published quantitative data (e.g. Templado 1984; Bonfitto et al. 1998; Solustri et al. 2002; Albano and Sabelli 2012) is hampered by the differences in sampling design and methods. Still, the 109 species here reported are only slightly less than the 139 in the methodologically most similar study on the meadow in Cabo de Palos (Murcia, south-eastern Spain) where sampling occurred across a broader depth range and on multiple years (Templado 1984). The Shannon diversity and Pielou’s evenness we recorded on both the leaf and rhizome strata are generally higher than the values recorded at Hvrgada Island in Croatia (Solustri et al. 2002), but lower than on the leaf stratum in the *Posidonia oceanica* meadow at Ischia Island, central Tyrrhenian Sea, at comparable depths (Idato et al. 1983).

The micrograzers *Jujubinus*, *Tricolia*, *Bittium* and Rissoidae are dominant in the leaf stratum like at *Posidonia oceanica* meadows in Spain (Templado 1984), in the Tyrrhenian Sea (Idato et al. 1983; Bonfitto et al. 1998; Albano and Sabelli 2012), in the Strait of Sicily (Accardo-Palumbo et al. 1992) and in the Adriatic Sea (Solustri et al. 2002). The rhizome stratum is still dominated by micrograzers, but has a more diverse trophic structure as it hosts also several filter-feeders (mostly bivalves), carnivores and ectoparasites; a typical pattern of *Posidonia oceanica* meadows (Templado 1984; Bonfitto et al. 1998; Solustri et al. 2002; Albano and Sabelli 2012). Despite some species move along the blade between day and night (so-called nychthemeral migrations (Russo et al. 1991)), all mentioned studies were conducted during the day making comparisons meaningful. Such comparisons suggest that the molluscan assemblage we inspected conforms to the expectations of healthy *Posidonia oceanica* meadows across the Mediterranean Sea.

In our study, the differences between seasons markedly varied with depth in the leaf stratum, where both Shannon diversity and evenness were greater in spring than autumn down to 10 m depth, but the opposite occurred at 15- and 20-m depth. These differences match those observed in Ischia Island in the Tyrrhenian Sea (Russo et al. 1991), where the structure of the leaf molluscan assemblage indeed varied with depth, but not with season. In the rhizome stratum, differences in Shannon diversity and evenness with season were minor and without a clear direction, comparable with the results from a very shallow water *Posidonia oceanica* meadow in the Alboran Sea (Urra et al. 2013). Also the proportions of the trophic guilds are similar between the two seasons in the Alboran Sea, whereas in our study microalgae herbivores and filter feeders showed in the rhizomes considerably greater abundance in spring than in autumn, possibly related to the greater availability of food in the water column during the spring phytoplankton bloom (Evans and Parslow 1985).

Of the four non-indigenous species found, only one, *Pinctada radiata*, is a long-term host of the Mediterranean Sea. It was recorded already in the second half of the nineteenth century, shortly after the opening of the Suez Canal (Vassel 1896). By now it has established populations all over the Mediterranean basin and reached as far as Spain (López Soriano and Quiñonero-Salgado 2019; Ballesteros et al. 2020; Png-Gonzalez et al. 2021) and the southern Adriatic Sea (Petović and Mačić 2018). *Septifer cumingii* was first recorded in the Mediterranean in 2001 (Albayrak and Çeviker 2001) and is now broadly distributed in the eastern Mediterranean (Katsanevakis et al. 2009; Albano et al. 2021a), whereas *Viriola* cf. *bayani* and *Isognomon* aff. *australica* are very recent introductions (Steger et al. 2018; Albano et al. 2021b), suggesting that not only the non-indigenous component is negligible, but that it is also of very recent acquisition.

Finally, yet importantly, in our samples there was a small but taxonomically and functionally diverse component of species endemic to the eastern Mediterranean. Endemic species at the scale of Mediterranean sub-basins were a small share of the diversity also in the Alboran Sea (Urra et al. 2013) and in the Tyrrhenian Sea (Albano and Sabelli 2012), showing on the one hand that *Posidonia oceanica* meadows host a quantitatively important component of the Mediterranean malacofauna, and on the other hand that this habitat is important for the survival of rare endemic species.

### *Posidonia oceanica* meadows: a refugium against climate warming and deoxygenation?

This healthy assemblage was in sheer contrast with our expectation of an impoverished area heavily affected by biological invasions. Indeed, there is increasing evidence of collapsing native assemblages in other parts of the eastern Mediterranean (Crocetta et al. 2013, 2020; Rilov 2016; Albano et al. 2021a) and Crete is among the most invaded sectors of the Greek seas (Zenetos et al. 2011) and of the Mediterranean basin (Galil 2012; Galil et al. 2014, 2018).

The Mediterranean Sea has a north-west to south-east gradient of increasing sea surface temperature making the eastern Mediterranean its warmest sector (Pisano et al. 2020). On top of this climatological pattern, the eastern Mediterranean is experiencing the highest rates of warming in the basin (Ozer et al. 2017; Pisano et al. 2020). Mediterranean biodiversity is mostly of temperate origin, being the result of the extreme climatic fluctuations of the Pleistocene and especially of its latest glacial-interglacial cycle (Sabelli and Taviani 2014). Consequently, most native species are poorly adapted to the increasing tropical conditions experienced by the basin. Ongoing warming pushes species beyond their thermal tolerance limits, making climate change the main driver beyond the native invertebrate collapse and the changes in fish abundances over the last 20 years recently recorded on the Israeli shelf (Rilov 2016; Givan et al. 2018; Albano et al. 2021a; Steger et al. 2021b). Although the increasing sea surface temperature trend has been recorded for Crete too, the study site currently lies across the 20.5 °C annual isotherm (Pisano et al. 2020). This is almost 1 °C higher than in the 1982–1993 period, but still 2 °C lower than the Israeli shelf where the most dramatic effects have occurred already (Pisano et al. 2020).

Climate warming causes local extinction in multiple ways (Cahill et al. 2012), but in the marine realm, temperatures that exceed the species physiological tolerance are considered a major driver for ectotherms, with species ranges advancing and retracting closely matching suitable isotherms (Sunday et al. 2012). Warming waters increase metabolic oxygen demand and at the same time contain less oxygen because of reduced oxygen solubility and the increased oxygen consumption (Breitburg et al. 2018). Such conditions cause reduced growth and survival (Vaquer-Sunyer and Duarte 2011; Breitburg et al. 2018; Pauly 2021). However, photosynthetic activity causes an oxygen supersaturation that can buffer the effects of warming by significantly increasing the thermal tolerance of marine ectotherm vertebrates and invertebrates (Giomi et al. 2019). Large *Posidonia oceanica* meadows cause an oxygen supersaturation up to 161% (Hendriks et al. 2014) and thus have the full potential to exert the same buffering effect on thermal tolerance as experimented by Giomi et al. (2019).

Such persistent diverse assemblages in a warming climate may contribute to reduce invasion success (Stachowicz et al. 1999). In marine ecosystems, this resistance is stronger in the subtidal and on hard substrates where both competitive and consumptive biotic resistance occurs (Kimbro et al. 2013). *Posidonia oceanica* meadows host extensive surfaces acting as hard substrates (e.g. the leaves and the rhizomes) and occur from subtidal down to 40 m depth (Gobert et al. 2006), making them a paradigmatic habitat for biotic resistance.

Despite that our results shall be deemed preliminary, they may suggest that healthy seagrass meadows constitute a unique refugium for native biodiversity from warming and increasingly invaded waters on the shallow shelf. Future studies may compare meadows with other substrates and measure oxygen production to build up a more robust framework. Still, this perspective would be particularly valuable in the vulnerable eastern Mediterranean that is already experiencing the climate-driven collapse of native biodiversity and reinforces the need to fully protect this habitat that is retreating all over the Mediterranean Sea (Telesca et al. 2015).

## Supplementary information

ESM 1 Quantitative data (XLSX 38 kb)

## Data Availability

Quantitative data are included in ESM1 and will be uploaded in OBIS.

## Declarations

### Sampling and field studies

The fieldwork has been conducted in compliance with all applicable regulations.
